# Metastasis: Genetics, Mechanism, and Diagnostic and Therapeutic Strategies

**DOI:** 10.1155/2012/390835

**Published:** 2012-12-30

**Authors:** Mehmet Gunduz, Esra Gunduz, Levent Beder, Davut Pehlivan, Omer Faruk Hatipoglu, Sushant Kachhap, Reidar Grenman

**Affiliations:** ^1^Departments of Medical Genetics and Otorhinolaryngology, Faculty of Medicine, Fatih University, Istanbul, Turkey; ^2^Department of Medical Genetics, Faculty of Medicine, Fatih University, Istanbul, Turkey; ^3^Department of Otorhinolaryngology, Wakayama Medical University, Wakayamashi, Wakayama, Japan; ^4^Department of Molecular and Human Genetics, Baylor College of Medicine, Houston, TX, USA; ^5^Department of Molecular Biology and Biochemistry, Okayama University, Okayama, Japan; ^6^Prostate Cancer/Genitourinary Program, The Sidney Kimmel Comprehensive Cancer Center, Baltimore, MD, USA; ^7^Department of Otorhinolaryngology, University of Turku and Turku University Hospital, Turku, Finland

Tumor masses are composed of heterogenic subpopulations of cancer cells, and cancer stem cell theory suggests that only a specific subpopulation of these cells has the ability to sustain cancer growth and metastatic activity, whereas all of the other cancer cells have only a limited growth potential or no growth potential at all. Based on this concept in the literature, to define the aggressiveness of a tumor, examination of the molecular characteristics of metastatic cell populations is a logical way to characterize cancer stem cells. 

 The general prognosis of patients with various cancer types has not improved significantly, despite major advances in early detection, surgical resection, and chemoradiation protocols. The poor outcome has mainly been attributed to local and distant lymph node metastasis as well as recurrence. The ability to assess or predict the presence of metastasis has significant prognostic relevance and treatment implications in the management of cancer. In order to decrease morbidity and mortality from cancer, it is necessary to gain a greater understanding of metastasis and define the molecular factors that contribute to this process. 

In this special issue, metastatic behaviour of several cancer types is reviewed. One of them is an aggressive tumor type, though its incidence is low. F. Tas presented the clinical outcome of a relatively large number of melanoma cases, and over 200 patients were followed up for metastasis and survival. Due to its aggressive character, more than 30% of the melanoma cases were already metastatic when it was diagnosed first time. Most common metastatic organs were lung, liver, bone, and brain. In another lethal cancer type, in pancreatic cancer, L. Farhana et al. examined the effect of adamantly-retinoid-related (ARR) molecules in 3 pancreatic cancer cell lines. They found that two different ARR compounds inhibited not only the pancreatic cancer cell lines but also their cancer stem-cell-like populations (CD44+/CD24+ cells), and this inhibition was through suppression of IGF1R and Wnt/BCatenin pathways. Induction of apoptosis in the pancreatic cancer cell lines by ARR compounds suggested novel therapeutic agents for this chemotherapy-resistant cancer type. In another interesting study for early diagnostics, C. Streckfus et al. compared the salivary protein components of two different breast cancer patients group, one is positive for Her2 and the other is negative for this marker, but they are all in the same tumor stages (II). They performed a proteomic analysis of the saliva from the two groups by LC-MS/MS mass spectrometer. 71 proteins among 188 comparative saliva proteins were differentially expressed. There were 34 upregulated proteins, while 37 proteins were downregulated. In conclusion, they suggested that salivary gland proteins may be a real-time in vivo follow-up marker for breast cancer progression. 

P. Ellis et al. examined angiogenesis in vulvar as well as breast Paget diseases. Using anti-Von Willebrand factor antibody, microvessel density was determined in Paget diseases of Vulva and Breast. Increased microvessel density was demonstrated in Paget's disease of the breast with DCIS and with carcinoma alone compared to Paget's disease of the breast alone, suggesting that neovascularisation is an important process in the development of Paget's disease of the breast. Metastasis to the central nervous system (CNS) remains a major cause of morbidity and mortality in patients with systemic cancers. Various crucial interactions between the brain environment and tumor cells take place during development of the cancer at its new location. In one of the current papers in this issue, G. Rahmathulla et al. summarized the principal molecular and genetic mechanisms that underlie the development of brain metastasis (BrM) from aspects of migration-related events and molecules such as epithelial mesenchymal transition, interaction with tumor stroma, e-cadherin catenin complex, integrins, matrix metalloproteinases, urokinase-type plasminogen activator, CAI1, and tumor colonization. By this way, the authors tried to increase knowledge of the metastatic process leading to better detection and treatment of brain metastases. 

METCAM, an integral membrane cell adhesion molecule (CAM) in the Ig-like gene superfamily, is capable of performing typical functions of CAMs, such as mediating cell-cell and cell-extracellular interactions, crosstalk with intracellular signaling pathways, and modulating social behaviors of cells. In this special issue, G. Wu et al. investigated many possible mechanisms mediated by this CAM during early tumor development and metastasis. METCAM-induced tumorigenesis has been studied in melanoma, prostate cancer, breast cancer, and ovarian cancer. In conclusion, they emphasized that preclinical trials using a fully humanized anti-METCAM antibody against melanoma growth and metastasis and using a mouse anti-METCAM monoclonal antibody against angiogenesis and tumor growth of hepatocarcinoma, leiomyosarcoma, and pancreatic cancer have been successfully demonstrated. Alternatively, small soluble peptides derived from METCAM may also be useful for blocking the tumor formation and tumor angiogenesis. 

M. Kuramoto et al. focused on peritoneal metastasis, which often arises in patients with advanced gastric cancer and is well known as a miserable and ill-fated disease. 

The authors suggested extensive intraoperative peritoneal lavage (EIPL) as a useful and practical adjuvant surgical technique for those gastric cancer patients who are likely to suffer from peritoneal recurrence. By this way, they tried to diagnose early and prevent peritoneal recurrences. 

Metastasis and its molecular steps are still unclear, and their identification is crucial for curing the cancer. Recent and future developments in molecular medicine will allow to better understand metastatic process and early diagnose as well as treat the patients in a better way. 

*Mehmet Gunduz* *Mehmet Gunduz* *Esra Gunduz* *Esra Gunduz* *Levent Beder* *Levent Beder* *Davut Pehlivan* *Davut Pehlivan* *Omer Faruk Hatipoglu* *Omer Faruk Hatipoglu* *Sushant Kachhap* *Sushant Kachhap* *Reidar Grenman* *Reidar Grenman*

